# Prevalence and risk factors associated with self-reported carpal tunnel syndrome (CTS) among office workers in Kuwait

**DOI:** 10.1186/1756-0500-5-289

**Published:** 2012-06-13

**Authors:** Sudha R Raman, Becher Al-Halabi, Elham Hamdan, Michel D Landry

**Affiliations:** 1 Fawzia Sultan Rehabilitation Institute, , Kuwait; 2 Doctor of Physical Therapy Division, Dept of Community and Family Medicine, Duke University, Box 104002, Durham, NC, 27708, USA; 3 Department of Physical Therapy, University of Toronto, Toronto, ON, Canada

**Keywords:** Carpal tunnel syndrome, Prevalence, Occupational exposure, Kuwait

## Abstract

**Background:**

The prevalence of carpal tunnel syndrome (CTS) is not well understood in many Arabian Peninsula countries. The objective of this study was to investigate the prevalence and factors associated with self-reported CTS in Kuwait.

**Findings:**

A cross-sectional, self-administered survey of CTS-related symptoms was used in this study. Multivariate logistic regression was also used to estimate adjusted odds ratios for factors of interest. Participants in this study were adult office workers in Kuwait (n = 470, 55.6% males), who worked in companies employing more than 50 people. Self-reported CTS was reported in 18.7% of the group (88/470). CTS was significantly associated with the following demographic factors: female gender, obesity and number of comorbid conditions. Self-identification of CTS was also associated with key symptoms and impairment in daily activities (e.g., wrist pain, numbness, weakness, night pain, difficulty carrying bags, difficulty grasping [Chi-Square Test for Association: *P* < 0.05 for all symptoms/activities]). However, symptoms such as wrist pain, weakness, and functional disabilities were also frequently reported among those who do not self report CTS (range: 12.1%–38.2%).

**Conclusions:**

Prevalence of self-reported CTS among office workers in Kuwait is 18.7%, and the risk factors for CTS in this population included female gender, obesity and number of related comorbidities. The frequency of symptoms in the sample who did not self report CTS suggest that CTS may be under-recognized, however further research is required to assess the prevalence of clinically diagnosed CTS.

## Background

Carpal tunnel syndrome (CTS) is a common musculoskeletal condition, and while diagnostic criteria for CTS can vary, the clinical profile typically includes a combination of clinical assessment of symptoms (e.g., numbness, tingling, night pain and paraesthesia), signs (e.g.,Tinel’s sign, Phalen’s sign) and/or nerve conduction velocity (NCV) testing of the median nerve across the carpal tunnel [1,2]. Prevalence estimates of CTS in the general adult population range from approximately 1% to 16% [3-6]. Little is known about the prevalence, costs and potential contributing factors of CTS in the Arabian Peninsula region, including Kuwait [7]. In Kuwait, high levels of overweight and obesity, diabetes mellitus, and cigarette smoking [8,9], and other known risk factors for CTS [10,11] may suggest that CTS is an emerging workplace and occupational health issue. The purpose of this study was to estimate the prevalence of self-reported CTS among office workers in Kuwait, and to identify risk factors associated with CTS in this population.

## Findings

## Methods

### Participants

A convenience sample of companies in Kuwait City employing 50 or more people were approached by the research team between June and July 2008 about participation in the study. All companies who were approached agreed to collaborate by allowing their individual employees to voluntarily participate in a questionnaire regarding CTS. In order to be included in this study, individual participants were required to be 20 years old or older, have an office job where their work duties and tasks were primarily related to administrative, computer and/or desk work, and be willing to complete the survey in English. In each location or participating company, a list of all employed office workers who met the inclusion criteria was obtained by the appropriate human resource department. Trained data collectors then described the study purpose and objectives to all potential participants, and at that time it was clarified that choosing to participate represented informed consent to participate in this study. Questionnaires were then distributed to all employees who agreed to participate. Ethics approval for this study was obtained through the Fawzia Sultan Rehabilitation Institute.

### Survey tool development

The data collection tool used in this study was a self-administered questionnaire that required approximately 15 minutes to complete. The questionnaire was developed specifically for the purpose of this study, and was pilot tested with 10 office workers employed by companies outside the study sample. Feedback from pilot testing resulted in clarification of the wording of 20 of 61 questions; and in all cases, changes were incorporated into the final questionnaire.

### Study measures and variables

There were three sets of questions in the survey (Additional file 1). The first set of questions was related to participant characteristics such as gender, age, height, weight, and nature of occupation (i.e. type of job). Body mass index (BMI: weight (kg)/height (m)^2^) was calculated and categorized according to World Health Organization (WHO) guidelines [12]. The second set of questions was related to CTS status. After reading a definition of CTS, participants who indicated ‘yes’ to the question, “Do you think you have Carpal Tunnel Syndrome?” were considered to have self-reported CTS. Participants self-reporting CTS were asked to indicate the duration, frequency, and severity of symptoms, the effect of their symptoms on work and daily activities disability, the effect of work on their development of CTS and whether they had been diagnosed with CTS by a health professional. The third set of questions was designed to test for the presence of CTS symptoms and risk factors via a series of questions for all participants regarding wrist pain, numbness, weakness, motor function, history of trauma, computer use and whether they suffered from any of 12 co-morbidities identified a priori from the literature [13-16]. Participants were also asked to indicate their smoking status, exercise frequency and perception of their general health.

### Data entry and analysis

Data were entered into a data file located on a password-protected computer in the research offices of Fawzia Sultan Rehabilitation Institute. Statistical analyses were conducted using SAS (SAS Institute Inc, Cary, North Carolina). Proportions were calculated to describe the outcome of interest (prevalence of self-reported CTS), demographic characteristics and CTS symptoms and disability. Symptom prevalence in individuals reporting CTS was compared to individuals not reporting CTS using Chi-square tests of association. The primary outcome variable of interest was self-reported CTS. Odds ratios and 95% confidence intervals (95% CIs) comparing individuals who reported CTS and individuals who did not, were calculated for factors of clinical interest (gender, age, BMI, self-rated health, previous wrist injury, computer use, number of co-morbid conditions, exercise frequency and smoking status). A best-fit logistic regression model of predictor variables for self-reported CTS was found using backward selection, starting from the full set of nine variables listed above. Adjusted odds ratios and 95% CIs for the variables remaining in the best-fit model were also calculated.

## Results

A total of 470 office workers from 12 different companies in Kuwait City participated in this study. Of those reporting, 55.6% were male, 60.7% were aged 20–30 years of age, and 60.6% were overweight or obese (Table 1).

**Table 1:** Sociodemographic and health characteristics of 470 office workers from 12 different companies in Kuwait City collected in June/July 2008

Characteristics	Total for Item	N (%)
Gender	466	
Male		259 (55.6)
Female		207 (44.4)
Age group (years)	466	
20–30		283 (60.7)
31–40		111 (23.8)
41–50		55 (11.8)
51+		17 (3.7)
Marital status	467	
Married		239 (51.2)
Single		210 (45.0)
Divorced/widowed		18 (3.9)
Work type	452	
Professional/white collar		444 (98.2)
Labor		8 (1.8)
Body mass index (BMI)	432	
Underweight (BMI: < 18.5)		8 (1.9)
Normal (BMI: 18.5 - < 25)		162 (37.5)
Overweight (BMI: 25 - < 30)		172 (39.8)
Obese (BMI: 30+)		90 (20.8)
Self-rated health	413	
Excellent/very good		174 (42.1)
Good		164 (39.7)
Fair/poor		75 (18.2)
Previous wrist injury	413	
Yes		84 (20.3)
No		329 (79.7)
Computer Use	397	
None		8 (2.0)
< 3 hours per day		115 (29.0)
> 3 hours per day		274 (69.0)
Number of comorbidities	381	
0		247 (64.8)
1		75 (19.7)
2		35 (9.2)
3+		24 (6.3)
Exercise frequency	369	
0 hour/week		134 (37.7)
1 hour/week		114 (30.9)
≥2 hours/week		116 (31.4)
Smoking status	362	
Yes		96 (26.5)
No		266 (73.5)

### Self-reported CTS

**Figure 1:**
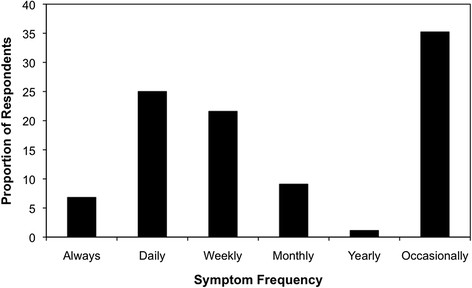
**Frequency of reported CTS symptoms amongst those who self-report CTS (n = 88).** Detailed Legend: Note that one individual did not indicate symptom frequency.

CTS was self-reported by 18.7% of the group (88/470). Of these individuals, over 30% reported that they experience the typical symptoms ‘daily’ or ‘always’ (Figure 1). Length of time since first noticing symptoms was less than one year for 37.5% of the sample, between 1 and 5 years for 43.2% of the sample, and longer than 5 years for almost 7% of the sample (12.5% did not indicate symptom duration). Only fifteen respondents indicated that they had taken sick leave because of their CTS. Of those self-reporting CTS, 71.6% (63/88) indicated that they believed their CTS was related to their work duties. Thirteen (14.8%) of those self-reporting CTS (or 2.8% of the entire sample) indicated that they had been previously diagnosed with CTS by a healthcare professional.

In bivariate analysis, self-reported CTS was significantly more likely to be found in females than in males, in individuals aged 31–40 years than in those aged 20–30 years, in obese individuals (BMI > 30), in those with a previous wrist injury, in those who rarely or never exercise, and in those who reported relevant comorbidities (Table 2). Due to small sample sizes for many of the investigated comorbidities, we focused on number of these comorbidities, rather than on the specific conditions. However, exploratory testing of the four most common conditions amongst the study population showed that CTS was more likely to be self-reported by individuals with depression (odds ratio, 95% CI: 3.1, 1.6-6.2), previous trauma to the cervical spine (4.2, 2.1-8.5), arthritis (2.5, 1.9-5.3) or diabetes (3.6, 1.5-7.6) than individuals without these individual conditions.

**Table 2:** Prevalence and odds ratios (OR) of self-reported CTS by sociodemographic and health factors of interest among office workers (Kuwait City, 2008)

	Total respondents	% (n) with CTS	OR	95% CI for OR	Adjusted OR*	95% CI for Adjusted OR*
Gender	456					
Male	254	13.4 (34)	1.0	Reference	1.0	Reference
Female	202	25.3 (51)	2.2	1.4, 3.5	4.7	2.1, 10.3
Age group (years)	456					
20–30	276	16.7 (46)	1.0	Reference		
31–40	109	27.5 (30)	1.9	1.1, 3.2		
41–50	54	16.7 (9)	1.0	0.5, 2.2		
51+	17	17.7 (3)	1.1	0.3, 3.9		
Body mass index	422					
Underweight	8	0.0 (0)	NE	NE	omitted	omitted
Normal	160	16.2 (26)	1.0	Reference	1.0	Reference
Overweight	167	15.6 (26)	1.0	0.5, 1.7	0.8	0.3, 1.9
Obese	87	29.9 (26)	2.2	1.2, 4.1	3.7	1.5, 9.6
Self-rated health	404					
Excellent/very good	170	10.0 (17)	0.4	0.2, 3.3		
Good	160	23.1 (37)	1.0	Reference		
Fair/poor	74	35.1 (26)	1.8	1.0, 3.3		
Previous wrist injury	405					
Yes	82	29.3 (24)	1.9	1.1, 3.3		
No	323	18.0 (58)	1.0	Reference		
Computer use	389					
None	8	0.0 (0)	NE	NE		
< 3 hours per day	111	14.4 (16)	1.0	Reference		
> 3 hours per day	270	21.1 (57)	1.7	0.9, 3.1		
Number of comorbidities	373					
0	244	10.2 (25)	1.0	Reference	1.0	Reference
1	70	28.6 (20)	3.5	1.8, 6.8	4.9	2.0, 12.3
2	35	31.4 (11)	4.0	1.8, 9.2	3.3	1.1, 9.7
3+	24	62.5 (15)	14.6	5.8, 36.8	14.9	4.8, 46.5
Exercise frequency	361					
0 hours/week	137	24.8 (34)	1.0	Reference		
1 hour/week	112	21.4 (24)	0.8	0.5, 1.5		
≥2 hours/week	112	9.8 (11)	0.3	0.2, 0.7		
Smoking status	355					
Yes	94	19.1 (18)	1.0	Reference		
No	261	19.2 (50)	1.0	0.5, 1.8		

Abbreviations: CI, confidence interval; CTS, carpal tunnel syndrome; NE, not estimable; OR, odds ratio.

*Estimated from multivariable logistic regression. Variables shown remained in best-fit model following backward selection starting from a model including all variables in the table; n = 245 respondents with no missing values.

The best-fit logistic regression with multiple predictors of self-reported CTS included a subset of the variables listed above. Female gender, BMI >30 and the presence of 1 or more of the selected comorbidities were independently associated with self-reported CTS (Table 2).

### CTS symptoms in study population

**Figure 2:**
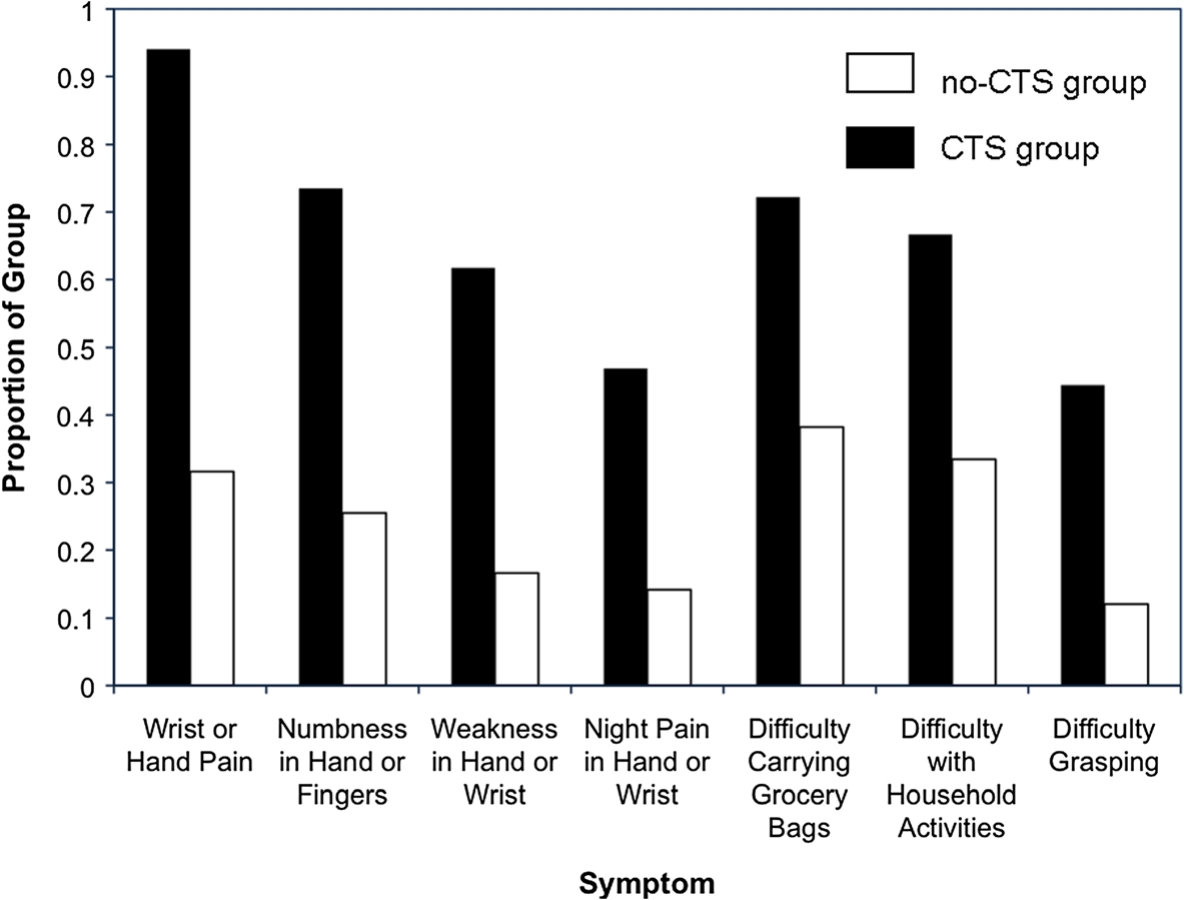
**Common symptoms of CTS reported by survey respondents.** Detailed Legend: Common symptoms of CTS reported by proportion of group self-reporting CTS (black bars) and by group not self-reporting CTS (white bars). Significant difference between groups for each symptom (Chi-Square Tests for Association: *P* < 0.05, for each).

Individuals self-reporting CTS were also more likely to indicate that they experience symptoms and difficulty with activities typically associated with diagnosed CTS than individuals who did not self-report CTS (Figure 2; *P* < 0.05 for each, Chi-square Tests of Association). Among those who did not report CTS, symptoms and difficulties were not uncommon (12%–38%; Figure 2). In contrast to the 4.5% (4/88) of individuals initially self-reporting CTS who indicated they were seeking treatment from a healthcare professional for their CTS, 68.4% (54/79) of those reporting CTS at the end of the survey indicated that they were now interested in seeking medical treatment.

## Conclusion

We report that the prevalence of self-reported CTS among office workers in Kuwait to be 18.7%, which is higher than has been reported in other non Arabian Peninsula countries [3-6]. The CTS prevalence estimate in this study may differ from previous studies due to real differences among office workers in Kuwait or the diagnosis of CTS in Kuwait. The fact that the definition of self-reported CTS in this study did not require current symptoms also may have contributed to higher estimate of self-reported CTS prevalence. Only 2.8% of the entire sample reported CTS diagnosis from a health profession, which supports the finding that prevalence of CTS by self-report tends to be much higher as compared to clinical exam and nerve conduction testing [5,13,14]. It would be expected that the estimated prevalence of CTS would decrease if clinical diagnosis and/or nerve conduction testing were used to validate the estimate; however, the magnitude of that decrease in this setting cannot be predicted. Similar to other research we found self-reported CTS to be more common in women than men [5,6], and that there is an association between self-reported CTS and obesity and relevant comorbidities [10,15]. As in recent reviews, computer use could not be shown as a definitive risk factor for CTS [16-18]. This study is limited by the use of a self-report measure rather than a clinical assessment, and by the use of a questionnaire that was not formally validated. Additional results were presented to provide context for the self-report prevalence estimate, including the proportion of those with diagnosed CTS and the prevalence of individual symptoms, some of which are used to diagnose CTS in the clinical setting (nocturnal sensory symptoms, numbness in the median nerve distribution, aggravating and alleviating factors). Reassuringly, the symptoms and functional impairments experienced by those who self-reported CTS were consistent with the clinical profile of CTS.

To our knowledge, this study presents the first estimate of self-report CTS prevalence in Kuwait and the first to suggest that a substantial proportion of individuals who did not self-report CTS describe high frequency of CTS symptoms. Given the dearth of information of the prevalence of CTS in this region of the world, we feel that self-report estimates of CTS can be taken in context as support for the need for further research into the prevalence of clinically diagnosed CTS and the possibility that CTS may be under-recognized in the general population in Kuwait. These findings, together with the documented high prevalence of factors associated with CTS in Kuwait [19-21] may signal that programs for the identification, prevention and intervention of musculoskeletal conditions such as CTS may have broad applicability across Kuwait. These data represent an important first step in recognizing what may be an emerging issue for occupational health across Kuwait.

## Abbreviations

BMI: Body Mass Index; CI: Confidence Interval; CTS: Carpal Tunnel Syndrome; NCV: Nerve Conduction Velocity; NE: Not Estimable; OR: Odds Ratio; WHO: World Health Organization.

## Competing interests

The authors declare that they have no competing interests.

## Authors’ contributions

SR contributed to the overall study design, data analysis, and writing drafts of earlier versions of this article. BH contributed to the overall study design, and lead the data collection and data entry components of this study, along with contributing to writing drafts of this manuscript. EH contributed to the overall study design, contributed to data collection and data entry, and contributed to writing drafts of this manuscript. MDL contributed to the overall study design, data analysis, and initiated the writing of all drafts of this manuscript. All authors read and approved the final manuscript.

## Supplementary Material

Additional file 1 **Appendix A.** Carpal tunnel syndrome study in office workers in Kuwait June 2008 Click here for file
