# Supplementary material for: Prevalence and risk factors associated with self-reported carpal tunnel syndrome (CTS) among office workers in Kuwait
Source: BMC Res Notes. 2012 Jun 13;5:289. doi: 10.1186/1756-0500-5-289 (PMC3403948; doi:10.1186/1756-0500-5-289)
Supplement: Additional file 1 — Appendix A. Carpal tunnel syndrome study in office workers in Kuwait June 2008 [file 1756-0500-5-289-S1.doc]

Appendix A

| **Carpal tunnel syndrome study in office workers in Kuwait June 2008** |
| --- |
|  |

**Part A: Background Information** Please provide an answer to all questions

A2. What is your gender?

 Male  Female

A3. How old are you?

 20-25  26-30  31-35  36-40  41-45
 46-50  51-55  56-60  60+

A4. What is your height? __cm Weight? __ kg Nationality?_________

A5. Are you?  Living alone  Living with relative/ friend

A6. What is your marital status?

 Married  Single  Divorced  Widow/ Widower

A7. Do you have any children?

Yes  No A7a. If **Yes** how many _________

A8. What type of work do you do?

 Professional (ex. Doctor, lawyer, nurse, teacher, computer/IT).

 White collar (ex. Office worker, lab tech).

 Skilled manual labor (ex. Electrician, plumber, carpenter).

 Unskilled labor (ex. Nanny, domestics etc).

A9. How many years of work experience do you have in your current job?

 0-5  6-10  11-15  16-20  21-25  25+

A10. What is your level of education?

 High School  Diploma  Bachelor  Master

 PhD  Others, specify __________

A11. Employment status

 Full-time employment  Part-time employment

**Part B: Prevalence of Carpal Tunnel Syndrome:** Please provide an answer to all questions.

**CTS** -a condition in which pain is felt in the wrist that radiates up the arm, with numbness felt in the thumb, index and middle fingers, and is increased by repetitive wrist movement.

B1. Do you think that you have Carpal Tunnel Syndrome (**CTS**)?

 Yes  No If you answered **'No'** please go to part **C**

B2. Have you experienced **CTS** symptoms in the last 24hours (pain or tingling sensation in the wrist/ numbness in the fingers)?

 Yes  No

B3. How often do you experience these symptoms?

 Always  Daily  Weekly

 Monthly  Yearly  Occasionally

B4. In general, how does **CTS** affect your life? Please circle one of the following statements that best describes your opinion.

No effect Severe effect

| 1 | 2 | 3 | 4 | 5 |
| --- | --- | --- | --- | --- |
| My CTS has no effect on my life | My CTS has a little effect on my life | My CTS has an effect on my life | My CTS has a moderate effect on my life | My CTS has a severe effect on my life |

B5. When did you first start to feel these symptoms?

 < 1 year  1-5 years  6-10 years  11-15 years  15+ years

B6. Do you think that **CTS** is related to your work duties?

 Yes  No

B7. Have you taken *sick leaves* off work because of **CTS**?

Yes No

B7a. If Yes, estimate how many days of sick leave you have taken off in the last year because of **CTS**?

 0-5  6-10  11-15  16-20  21-25

 26-30  31-35  36-40  41+

B8. Are you paid a salary when you take a sick day because of your **CTS**?

 Yes  No

B8a. Have you applied to any work induced disability compensation because of CTS?

 Yes  No

B9. Did you have to modify your work duties or transfer to other departments because of **CTS**?

 Yes  No

B9a. If **Yes,** did you move to a department of work duties with *less wrist* involvement?

 Yes  No

B10. How has work affected your **CTS**? Choose **one** statement **only**.

No effect Severe effect

| 1 | 2 | 3 | 4 | 5 |
| --- | --- | --- | --- | --- |
| My work has not affected my CTS | My work has slightly affected my CTS | My work has had some affect my CTS | My work has moderately affected my CTS | My work has severely affected my CTS |

B11. Have you ever been diagnosed with **CTS** by a health care professional?

 Yes  No

B11a. If Yes, which health professional made the diagnosis?

 Orthopedic  Neurologist

 Physiotherapist  Others, Specify__

**Question B11b (containing value of other health prof) was omitted during data entry revision to match data and for statistical insignificance. Done on 10/10/2008 by Bisher**

B12. Are you currently seeing a health care professional for treatment of your **CTS**?

 Yes  No

B13. Did you receive any treatment, if Yes, What kind of treatment have you had?

NO, I haven’t received any treatment.

Medications such as inflammatory drugs, painkillers

 Physiotherapy, manipulation, Hydrotherapy

 Conservative (Splinting, acupuncture)

 Any other treatment, please specify________________

**Question B13a (containing value of other treatment) was omitted during data entry revision to match data and for statistical insignificance. Done on 10/10/2008 by Bisher**

B14. Have you had surgery to treat CTS?

 Yes  No

B14a. Was it successful?

 Yes  No

**Part C:** Please provide an answer to all questions

C1. Have you ever had a hand or wrist pain?

 Yes  No

C1a. How often do you have hand or wrist pain during the day time?

 Never  Once or twice a day  3/5 times a day  >5 times a day  Constant

C1b. How long on average does an episode of pain last during the daytime?

 Never  <10 min  10-60 min  > 60 min  Constant

C1c. Does this pain increase at night?

 Yes  No

C2. Have you ever injured your wrist accidentally?

 Yes  No

C2a. If Yes, then was it followed by any tingling or numbness then?

 Yes  No

C2b.How often did you wake up due to pain in your hands in a typical night during the past two weeks?

 Never  Once  2/3 times  4/5 times  >5times

C3. Do you have numbness any (loss of sensation) in your hand or fingers?

 No  Mild  Moderate  Severe  Very severe

C3a. How severe is the numbness (loss of sensation) or tingling at night?

 No  Mild  Moderate  Severe  Very severe

C4. Do you have weakness in your hand or wrist?

 No  Mild  Moderate  Severe  Very severe


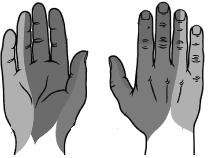

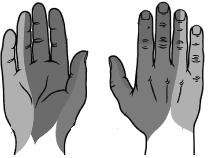


C5. Mark the area of most pain

or numbness on your hand

on the respective figure?

C6. Do you have difficulty grasping and using small objects such as keys or pencils?

 No  Mild  Moderate  Severe  Very severe

C7. Do you use computers? Yes No

C7a. If yes, how many hours do you use computers daily?

<1Hr 1-2 Hr 2-3 Hr 4-5 Hr 5-6 Hr > 6 Hr

C7b. During your use of computer, which item does use the most?

 Key board  mouse

C7c. Do you take breaks during work hours involving typing, for how long?

None 5min <15min 15-30min >30

C7d.While typing, in which position are you **MORE** likely to align your wrist? Choose one.

**
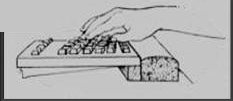

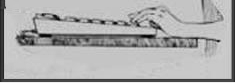
**

1

2

**Part D: Associated risk factors with CTS** Please answer all questions

Please mark the difficulty level you face while performing the following activities:

| Level of difficulty | | None | Mild | Moderate | Severe | Cant at All |
| --- | --- | --- | --- | --- | --- | --- |
| D1 | Writing |  |  |  |  |  |
| D2 | Buttoning of shirt |  |  |  |  |  |
| D3 | Holding a book while reading |  |  |  |  |  |
| D4 | Gripping a telephone handle |  |  |  |  |  |
| D5 | Opening jars |  |  |  |  |  |
| D6 | Performing household activities |  |  |  |  |  |
| D7 | Carrying grocery bags |  |  |  |  |  |
| D8 | Bathing and dressing |  |  |  |  |  |

Do you, or have you ever had, one or more of the following medical conditions?

|  | Condition | Yes | No |  | Condition | Yes | No |
| --- | --- | --- | --- | --- | --- | --- | --- |
| D9 | Diabetes |  |  | D15 | Hypothyroidism |  |  |
| D10 | Arthritis |  |  | D16 | Depression |  |  |
| D11 | Acromegaly |  |  | D17 | Amyloidosis |  |  |
| D12 | Gout |  |  | D18 | Multiple sclerosis |  |  |
| D13 | Tuberculosis |  |  | D19 | SLE |  |  |
| D14 | Renal failure |  |  | D20 | Trauma to cervical spine |  |  |

D21. For **females** only, did these symptoms of pain in the wrist or numbness in the fingers increase during:
Pregnancy Use of oral contraceptives  Post menopause

D22. Do you smoke? Yes No

D22a. If yes, how would assess your level of smoking?

Light smoker Moderate smoker Heavy smoker Very heavy smoker

D23. How many hours do you exercise weekly?

Never 1Hr 2-3 Hrs >3Hrs

D24. How would you assess your overall health?

Poor Fair Good Very Good Excellent


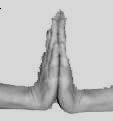
D25. Choose the best description of your feeling while aligning your hands in the position shown in the figure?

 Pain

 Pain + numbness of the fingers

 None

D26.After reading and filling out this questionnaire, do you think you might be having **CTS**?

Yes No

D27. If **Yes**, do you plan to seek any medical care to treat this condition?

Yes No

D28. If **No**, why do you think you will not be going to seek any medical help?

I don’t think it necessary

I don’t have time

I will try to manage my pain problem

I will try to change my life style to manage the condition

D29. Is there anything else that you would like to tell us about wrist pain in Kuwait?

**THANK YOU FOR YOUR PARTICIPATION**
